# Mental fatigue prediction during eye-typing

**DOI:** 10.1371/journal.pone.0246739

**Published:** 2021-02-22

**Authors:** Tanya Bafna, Per Bækgaard, John Paulin Hansen

**Affiliations:** 1 Department of Management, Technology and Economics, Technical University of Denmark, Kongens Lyngby, Capital Region of Denmark, Denmark; 2 Department of Applied Mathematics and Computer Science, Technical University of Denmark, Kongens Lyngby, Capital Region of Denmark, Denmark; University of Tübingen, GERMANY

## Abstract

Mental fatigue is a common problem associated with neurological disorders. Until now, there has not been a method to assess mental fatigue on a continuous scale. Camera-based eye-typing is commonly used for communication by people with severe neurological disorders. We designed a working memory-based eye-typing experiment with 18 healthy participants, and obtained eye-tracking and typing performance data in addition to their subjective scores on perceived effort for every sentence typed and mental fatigue, to create a model of mental fatigue for eye-typing. The features of the model were the eye-based blink frequency, eye height and baseline-related pupil diameter. We predicted subjective ratings of mental fatigue on a six-point Likert scale, using random forest regression, with 22% lower mean absolute error than using simulations. When additionally including task difficulty (i.e. the difficulty of the sentences typed) as a feature, the variance explained by the model increased by 9%. This indicates that task difficulty plays an important role in modelling mental fatigue. The results demonstrate the feasibility of objective and non-intrusive measurement of fatigue on a continuous scale.

## Introduction

Acute mental fatigue is revealed as a critical issue across the general working population, as work shifts from being physically to mentally challenging [1, 2]. Acute mental fatigue is caused by sustained cognitive processing over a period of time [3]. We use acute mental fatigue interchangeably with mental fatigue for the rest of this paper. Fatigue, physical as well as mental, is a relevant problem especially for people with neurological disorders like Amyotrophic Lateral Sclerosis (ALS), Cerebral Palsy (CP), or Multiple Sclerosis (MS) [4–6], as a result of increased fatigability [7]. People with neurological disorders, having restricted use of limbs and reduced oral abilities, are increasingly using an augmented and alternative system with eye-tracking to work and communicate. Fatigue, which we consider to incorporate mental fatigue, can cause reduced quality and quantity of communication [5]. In the current study, we tested the feasibility of mental fatigue prediction on a continuous scale with healthy volunteers during an eye-typing task.

Several studies have explored mental fatigue detection, caused by a prolonged cognitive task, from features measured using eye-tracking, such as pupil diameter, blinks and saccades. Tonic changes in pupil diameter are linked to mental fatigue and arousal via neural activity in locus coeruleus and as mental fatigue increases, baseline-related pupil diameter is expected to reduce [8]. Blink features such as blink frequency, blink duration and blink interval have been shown to be sensitive to increasing time-on-task [9, 10]. Bursts of blinks is another phenomenon studied, where increasing mental fatigue is accompanied by increase in blink bursts [11]. Eye movement features derived from saccades—rapid eye movements between gaze positions—have also been associated with mental fatigue. Saccades have been found to get shorter and faster as individuals get more fatigued [12, 13].

Most experimental setups investigating mental fatigue manipulate the task duration to induce fatigue and analyse variation in fatigue with time-on-task [8, 14–18]. Borragán has shown that while time-on-task plays a role in generating mental fatigue during continuous cognitive processing for an extended period of time, cognitive load, or the demand for allocation of mental resources to the task, is also an important factor [19]. Previous research has explored variations to the theory on mental fatigue caused by cognitive load, and they emphasise that mental fatigue is imposed from individual perception of high task demands, rather than high cognitive load per se [20, 21]. Pattyn et al. have extended this theory and created theoretical models that place an important role on the perception of effort and its effect on mental fatigue [22]. However, the influence of the cognitive load on fatigue measurement using eye-tracking features has not been explored.

Eye-tracking based psycho-physiological signals have been used to classify mental fatigue in healthy individuals [17, 18]. These papers classify fatigue into two mental states—fatigued and alert. However, we hypothesise that fatigue assessment have more levels than just the binary states. The bases for this hypothesis are that (1) mental fatigue increases in an accumulative process [23] and (2) mental fatigue questionnaires that have been used reliably in the medical field use non-binary scales to determine fatigue, rather than specify a threshold to classify the user as fatigued or alert [24–26]. Moreover, methods to counteract fatigue, such as taking a break [27, 28], or monitor health [18] could be improved further and personalised to the level of fatigue. Tracking mental fatigue with a higher granularity can be useful to systematically explore other ways to counter the problem of fatigue. Furthermore, with the ubiquitous and non-intrusive nature of eye-tracking, mental fatigue detection could help to improve the quality of life for people with neurological disorders as well as the general working population.

In the present study, cognitive processing during a task of eye-typing was used to induce mental fatigue, which was classified into six increasing levels of self-evaluated mental fatigue. Eye-typing is a known eye-based interactive task. The most common method for eye-typing is to fixate on each key on an on-screen keyboard for a certain amount of time (known as dwell-time), until the key is selected [29]. The cognitive processing on the eye-typing task in the present experiment was induced by asking the participants to memorise sentences of varying difficulty and eye-type them from memory, thus eliciting cognitive load on the participants. We identified the eye-based features most useful for the assessment of mental fatigue. Since the participants were not restricted in their movement, we decided to also study their posture and its relation to fatigue, based on known relations of increased postural variations during low arousal periods in tasks [30], and observations of participants lowering themselves in the chair as the experiment progressed. Finally, we also studied performance measures commonly measured using eye-typing—typing speed, error rate, attended but not selected rate (ANSR) for keys and read text events ratio (RTE) [31]. ANSR and RTE are associated with the error rate during typing and accuracy of the gaze-typing system [32]. Since most of the above physiological measures are also commonly investigated when studying cognitive load [33–37] and mental fatigue is affected by cognitive load, in this paper, we will attempt to explain the impact of the relationship between cognitive load and fatigue on the features studied.

## Materials and methods

### Participants

Nineteen healthy volunteers (nine males, 10 females, Age: 25.5 *years* ± 2.38), all university students, participated in the study. None of the participants had photosensitive epileptic seizures or a history of a brain disorder. The Scientific Ethics Committee for the Capital Region in Denmark approved the study protocol (approval number H-18052072). All participants provided written and informed consent to participating in the study, and they received a gift card worth 500 DKK on finishing the experiment. One participant did not complete the study and was omitted from the analysis.

### Experimental design

**Fig 1:**
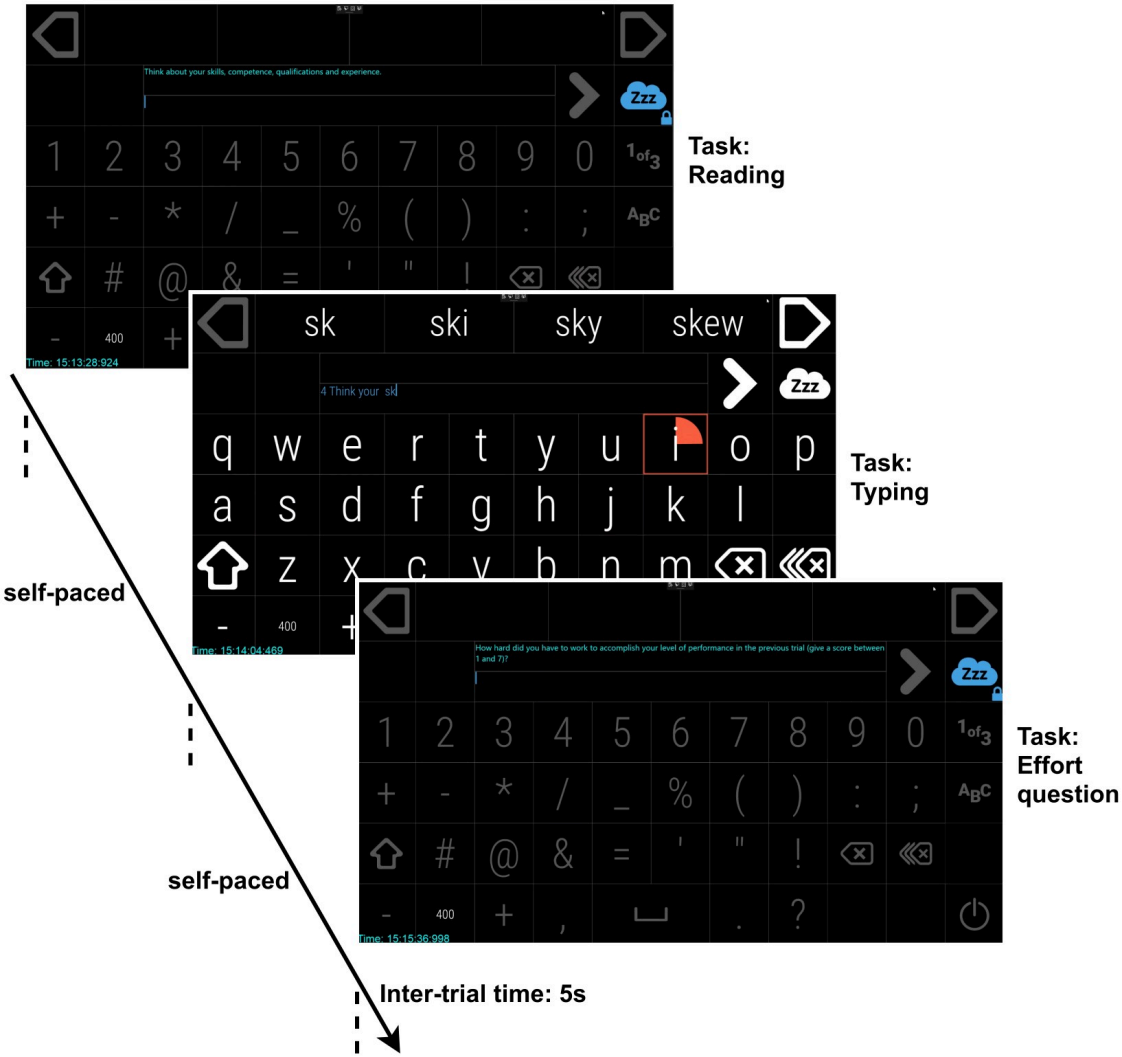
Tasks and their order during a trial—reading and memorising an example sentence *Think about your skills, competence, qualifications and experience*., typing from memory and reporting the perceived effort during the completed task. The blue text during the reading task displays the sentence to be typed. When the typing task starts, the sentence to be memorised disappears.

Each participant performed the experiment during four different days. Each day, two sessions were performed in one seating. Each session was composed of five typing-from-memory trials, which involved reading and memorising a sentence, and typing it from memory using eye-typing (Fig 1). The source of the sentences was the Leipzig corpus [38], and the readability score—Lasbarheitsindex (LIX) score [39]—was used to define the level of difficulty. For simplicity, two levels of difficulty were established based on the LIX score—easy, with a LIX score of less than 30 and difficult, with a LIX score of more than 60. During an easy session, the typing-from-memory trials involved five easy sentences, and five difficult sentences were applied during the difficult session. The order of easy and difficult sessions was balanced for each participant. Between each trial, 5 s of break time was provided to the participants, to allow the phasic arousal to return to the baseline [15].

On the first day, after the participants signed the consent form, they read the instructions on the experiment and the typing procedure. This was followed by a practice session. The experiment was performed on an on-screen keyboard Optikey [40] using the eye-tracker Tobii Eye Tracker 4C. The experiment room had lighting of 25-60 lux at the computer screen.

**Fig 2:**
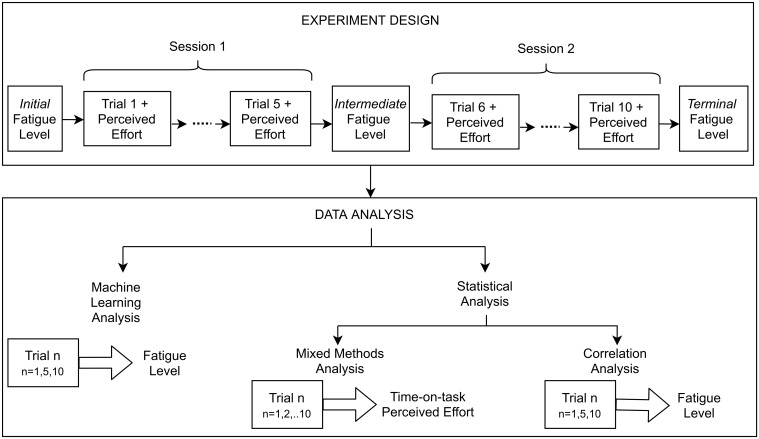
Design of the experiment during one day consisted of two sessions and a total of ten trials. Data Analysis was divided into two parts—prediction of the fatigue level, correlation analysis and linear mixed model of the data for effects of time-on-task and perceived effort. Trials 1,5 and 10 were used as representative of the users’ state before and after sessions 1 and 2, respectively, and employed for the prediction and correlation analyses. All trials were used for the linear model.

At the end of every trial, the participants answered the *effort* question from NASA—Task Load Index questionnaire (NASA-TLX) on a seven-point Likert scale, by selecting a number using eye-tracking on the on-screen keyboard, in response to the question on the screen, thereby reporting the perceived effort during the trial. Before starting the experiment each day and after every session, a question on the subjective level of fatigue on a seven-point Likert scale was orally answered by the participants [16]. The experiment design is shown in Fig 2.

Native danish speakers performed the test in Danish, and everyone else performed it in English. Ten participants performed it in English.

### Features

The features computed were divided into three groups—performance-based features, eye-based features and self-reported measures. They are listed in Table 1, with descriptions of each feature.

**Table 1:** Data category and features used to analyse data, and their definitions.

Data Category	Feature (units)	Computation method
**Performance features**		
Performance	Typing speed (WPM)	Ratio of number of words typed, where one word was counted as five characters, including space key, and total trial time.
	Corrected error rate (%)	Ratio of number of backspace selections to total characters typed in a trial
	Uncorrected error rate (%)	Equally weighted character and word error rate for a trial, where error rate is the ratio of uncorrected character/word errors to total characters/words typed
	Read text events ratio	A ratio of time spent reading the text typed to the trial typing time
	Attended but not selected rate	Ratio of time spent on keys attended, but not selected, to total selected keys in the trial
**Eye-based features**		
Pupil	Baseline-related pupil diameter (cm)	Absolute pupil diameter computed during 0.300 s period of non-interpolated time at the end of 5 s of inter-trial time, for every trial
Blink	Blink frequency (Hz)	Ratio of number of blinks to the trial time
	Blink duration (s)	Average duration of blinks during the trial
	Blink interval (s)	Average time interval between consecutive blinks in a trial
	Blink burst ratio	Ratio of the blink burst events to the blink frequency
Eye Movement	Saccade amplitude (°)	Distance travelled during saccades in a trial
	Saccade duration (s)	Average duration of saccades during the trial
	Saccade peak velocity (°/*s*)	Mean of the highest velocity during a saccade, denoting the fastest movement within the saccade, during a trial
Posture	Eye height (cm)	Mean relative vertical position of the eye in a trial
**Self-reported measures**		
Cognitive load	Perceived effort	Subjective evaluation of the *effort* question from NASA-TLX on a scale of 1 to 7
Mental fatigue	Fatigue level	Subjective evaluation of the question—how tired are you at the moment on a scale of 1 to 7?

WPM denotes words per minute

Eye-tracking data, obtained using the Tobii Pro software development kit, was filtered by removing invalid data (data points from the Tobii Eye Tracker 4C that remained constant in all the data fields) and interpolating spontaneous blinks, defined as missing data for a continuous duration of range 0.075-0.500 s. The pupil data was filtered by removing 0.200 s of pupil data before and after the blinks and replaced with a linear interpolation of the pupil diameter. This was followed by application of a hampel filter [41] with removal of outliers larger than 3 standard deviations of the averaged data over 5 samples around the current data sample. The pupil diameter from the right and left eye were combined using a weighted average, with weights computed from the inverse of the standard deviation of 25 samples until the current data sample. A Hidden Markov Model was used to label saccades, fixations and noise [42, 43]. Fixations of duration less than 0.100 s and saccades of amplitude less than 0.5° were labelled as noise. Furthermore, successive fixations separated by less than or equal to 0.075 s, and the centroids of which were less than 0.5° away, were merged.

Blink bursts were identified as two or more blinks occurring within a span of 2 s. The feature eye height was computed from the vertical position of the eye. Difference between the eye height during the trial and at the beginning of the day was used to define the feature.

Pearson correlation between right and left pupil diameter was used to determine the quality of the data. Sessions with a correlation value lower than a threshold of 0.75 were removed from data analysis of the features.

Self-reported measures were used for subjective evaluation of the cognitive load and mental fatigue. The *effort* question from NASA-TLX was selected to focus on the perception of the effort applied by the participants on the tasks. A single-item measure using the word *tired* was used to define the fatigue level [16, 44]. The experiment required cognitive processing, and did not involve any physical activity. Moreover, the participants were not restricted in their movement, and thus the fatigue level was assumed to define mental fatigue.

### Data analysis

#### Analysis of self-reported measures

Two types of manipulation check were implemented, based on the perceived effort of each trial and the fatigue level obtained after every five trials.

The perceived effort was examined for the effects of the the objective task difficulty, session number, day number and language using linear mixed models (LMM).

Fatigue level was examined to find out if performing the cognitive tasks had an effect. Initial fatigue level was recorded before the experiment started, intermediate fatigue level after session 1 (after five trials) and terminal fatigue level after session 2 (after ten trials). Wilcoxon-ranksum test was performed in the three following sections, analysing (1) difference between the intermediate and initial fatigue level, (2) difference between the terminal and intermediate fatigue level and (3) difference between the terminal and initial fatigue level, and if they varied from 0. Additionally, fatigue level was analysed for the effect of the objective task difficulty, day number and time of evaluation. To preserve the independence of the fixed variables objective task difficulty and time of evaluation, the analysis was performed in 3 separate sections, analysing (1) difference between the intermediate and initial fatigue level for the effect of objective task difficulty of session 1, day number and language, (2) difference between the terminal and intermediate fatigue level for the effect of objective task difficulty of session 2, day number and language and (3) difference between the terminal and initial fatigue level, to examine the effect of the order of the objective task difficulty (comparing easy followed by difficult session to difficult followed by easy session), day number and language. LMM was fitted to the difference in fatigue level for each of the above cases.

#### Machine learning analysis: Prediction of the fatigue level

Four models were tested for the prediction of the fatigue level—adaBoost regressor with regression trees (RT), random forest regression (RFR), partial least squares regression (PLS) and support vector regression with bagging (SVR). The machine learning methods were implemented using the Scikit-learn library (version 0.22.1) in Python (version 3.6.10). Hyperparameters for all four models were optimised using grid search and 5 repetitions of 5-fold cross-validation in the Scikit-learn library.

The training and testing data was normalised to unit Euclidean length. The mean absolute error (MAE) from 5 repetitions of 5-fold cross-validation was used as the primary metric, with 80% of the data as training data, to compare the performance of the models. We compared with a random predictor based on Monte Carlo simulations of the target variable, where the target variable had the same distribution as the fatigue level data collected in the study; the MAE computed using this simulated data was used to establish the baseline prediction performance of the fatigue levels.

To identify features that generated the best performance of the models, feature selection through recursive evaluation was performed and compared to the models generated with all the features. The model with the lowest MAE was chosen as the final model. To the feature combination selected from this step, objective task difficulty was added as a feature and the model results of MAE and explained variance were compared to the original model without the objective task difficulty. Feature importance was further computed to explain the importance of the various selected features.

Up to now, the models were applied in an subject-independent cross-validation setting, where the data from all subjects was pooled together to train the model, and the testing data was composed of all subjects. As a last step, the final model was applied in a cross-subject setting and MAE results from leave-one-subject-out cross-validation (LOSOXV) were discussed. These results would hint at the robustness of the model and show whether the inter-subject differences in the features selected are greater than the intra-subject differences.

#### Statistical analysis: Effect of time-on-task and perceived effort on the features

To better understand the working of the machine learning models, the effect of perceived effort and time-on-task on the features was analysed using mixed methods analysis. The entire data analysis is depicted, along with the experiment design, in Fig 2.

The fixed effects used were of two types—factors, which was language (Danish/English), and numerical variables comprising of perceived effort, day number (with four increasing levels) and time-on-task (with 10 increasing levels). Perceived effort (with seven subjectively defined levels), replaced the objective task difficulty (with two objectively defined levels) as a fixed effect, as the features were expected to be more sensitive to the perceived effort. Random intercepts were used to model the random effects of the within-subject variability and random slopes for the perceived effort were added to the model when found to be significant using the step function from lmerTest package and when the final model converged. Significance was set at 0.05.

Packages lmerTest (version 3.1.2) [45] and lme4 (version 1.1.23) [46] in R (version 4.0.2) [47] were used to implement the models, and effect sizes were computed using the package r2glmm (version 0.1.2) [48], which used the Nakagawa and Schielzeth approach [49]. The p-values were computed using the Satterthwaite degrees of freedom. Additional post-hoc analysis was performed using the package multcomp (version 1.4.13) [50] and Bonferroni correction for the p-values.

#### Statistical analysis: Correlation between fatigue level and the features

To assess the role of subjective reports of the fatigue level in explaining the machine learning models, Pearson correlations between the fatigue levels and the features from the trial numbers 1, 5 and 10 were performed. Significance was set at 0.05.

## Results

Each of the 18 participants performed the experiment on four days in total, with 10 trials on each day. This resulted in a total of 720 trials. Due to a deviation in settings, seven extra trials were performed, and they were removed from analysis if no self-reported measure was obtained for the trial. Self-reported measure perceived effort was obtained for 704 trials, which were all used for its analysis. The correlation between the right and left pupil diameter was below 0.75 in 15 sessions, which were removed due to increased noise. Furthermore, trials where data from any feature was missing were also removed. This resulted in a final selection of 623 trials, such that each participant had at least 10 trials. Data for each trial consisted of the performance and eye-based features computed from Table 1 and the perceived effort by the participant.

The data from trials 1, 5 and 10 on the four days for 18 participants amounted to 216 trials. Fatigue level data was obtained for 209 of the trials, which were all used for the manipulation check. Trials from noisy sessions and with missing data were removed, such that each participant had at least six trials and each fatigue level had at least five data points. One participant with two trials and the trial with fatigue level 7, which had only one data point, were removed resulting in 183 remaining trials. The data from these trials and the fatigue levels were used for machine learning, to predict fatigue level on a six-point Likert scale and for correlation analysis.

### Analysis of self-reported measures

The perceived effort was examined, to determine if it showed an effect of the objective task difficulty in the experiment. The marginal mean of perceived effort showed a difference of the objective task difficulty (Easy: 2.95, 95% CI [2.57, 3.33], Difficult: 4.72, 95% CI [4.34, 5.10]). The perceived effort decreased each day by 0.339 (SE = 0.045) and increased during the second session by 0.415 (SE = 0.142). Using linear mixed models, we found that the objective task difficulty had an effect on the perceived effort (*χ*^2^(2) = 262.88, *p* < 0.001, *η*^2^ = 0.012). The effect of the day number was significant (*χ*^2^(1) = 54.849, *p* < 0.001, *η*^2^ = 0.060) and so was the session number (*χ*^2^(2) = 8.651, *p* < 0.05, *η*^2^ = 0.010). There was an interaction between the session number and objective task difficulty and the perceived effort for the easy session reduced during the second session by 0.469 (SE = 0.200), but the effect was not significant after multiple comparisons.

**Fig 3:**
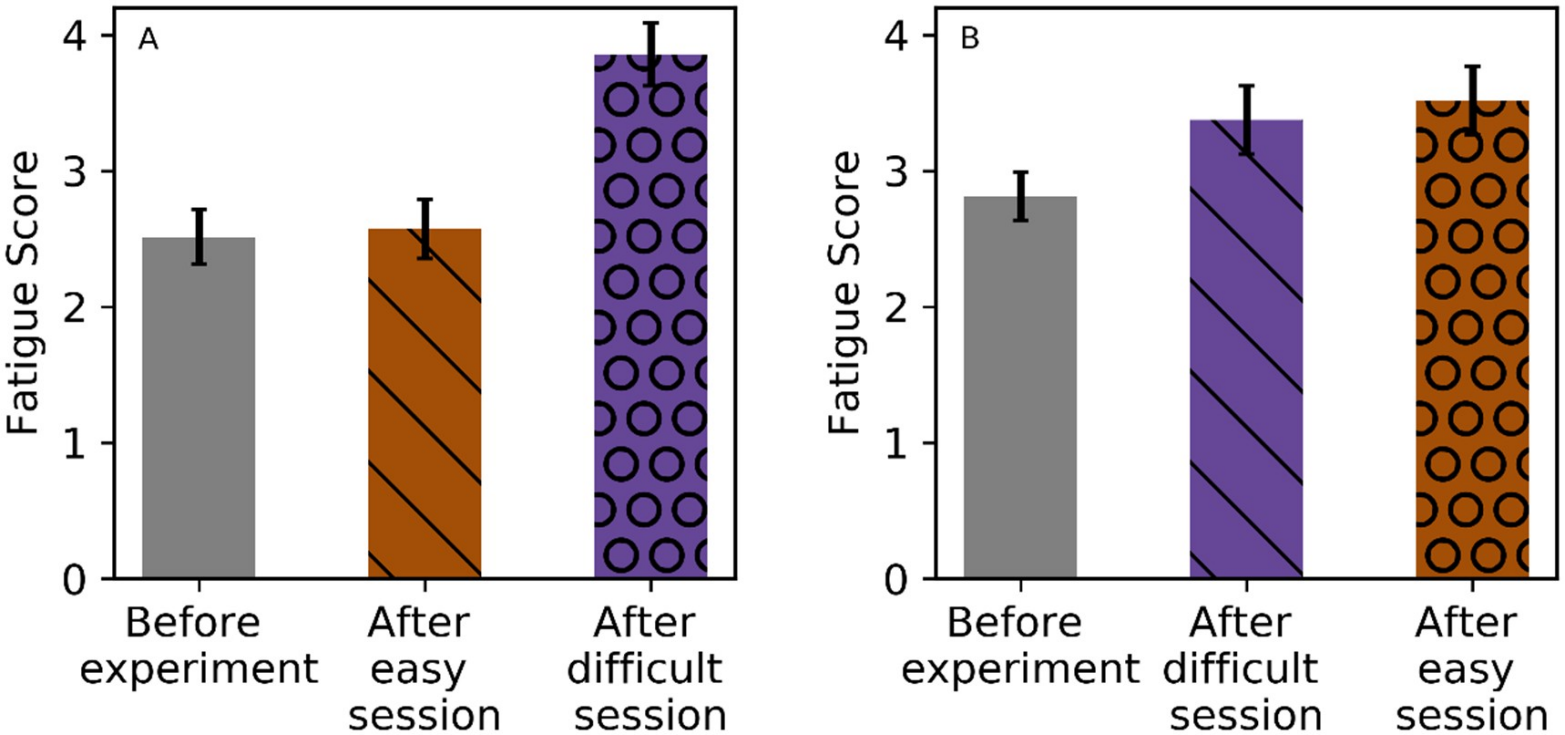
Subjective fatigue levels—Initial (without pattern), intermediate (lined) and terminal (dotted), for the two different orders of easy (orange) and difficult (purple) sessions. Error bars indicate standard error.

Fatigue level was investigated in 3 parts. (1) Initial and intermediate fatigue level: the mean of the difference between intermediate and initial fatigue level was 0.299 (95% CI [0.002, 0.596]). Wilcoxon-ranksum test revealed significant difference between initial and intermediate fatigue levels (V = 421, p = 0.036). Marginal means of fatigue level showed an increase in the intermediate from initial fatigue level by 0.062 (95% CI [-0.358, 0.481]) after an easy session and by 0.556 (95% CI [0.117, 0.996]) after a difficult session. Comparison of initial and intermediate fatigue levels showed no effect of the objective task difficulty of the first session using LMM (p = 0.084). (2) Intermediate and terminal fatigue level: The mean of the difference between the terminal and intermediate fatigue level was 0.714 (95% CI [0.331, 1.098]). Wilcoxon-ranksum test showed a significant increase in the terminal fatigue level compared to intermediate fatigue level (*V* = 914, *p* < 0.001). Marginal means show increase in the terminal from intermediate fatigue level after an easy session by 0.139 (95% CI [-0.393, 0.670]) and after a difficult session by 1.286 (95% CI [0.755, 1.82]). Comparison of intermediate and terminal fatigue levels using LMM demonstrated an effect of the objective task difficulty of the second session (*χ*^2^(1) = 9.966, *p* = 0.002, *η*^2^ = 0.131). (3) The mean of the difference between terminal and initial fatigue level was 1.045 (95% CI [0.686, 1.405]). Wilcoxon-ranksum test revealed a significant difference between terminal and initial fatigue level (*V* = 900.5, *p* < 0.001). Marginal means of fatigue level showed that easy session followed by difficult resulted in an increase in terminal from initial fatigue level by 1.353 (95% CI [0.836, 1.870]), and difficult followed by easy session resulted in an increase in terminal from initial fatigue level by 0.717 (95% CI [0.170, 1.26]). Comparison of initial and terminal fatigue levels revealed no difference in the order of the sessions using LMM (p = 0.061). Fig 3 depicts the subjective fatigue level recorded during the experiment.

### Machine learning analysis: Prediction of the fatigue level

Monte-carlo simulations resulted in a baseline MAE of 1.487. All the machine learning models were compared to this baseline error. The cross-validation results on MAE computed from the 80% training data and 20% testing data, for models generated on all features and for features selected using recursive feature elimination are given in Tables 2 and 3. Both RFR models explained high variance in the data while resulting in a low MAE. Based on the consistent results, RFR with recursive feature elimination was selected as the best performing model over the RFR model using all features (20% testing data MAE = 1.157), due to the use of fewer features. This model was a 22% improvement from the baseline performance.

**Table 2:** Model validation based on 80% training data.

Machine Learning Model	RFR	PLS	SVR	RT
**All features**				
**MAE**	0.943	1.022	0.952	1.069
**explained variance**	23.069%	13.248%	4.439%	13.604%
**Selected features**				
**MAE**	0.979	0.991	0.939	0.959
**explained variance**	20.929%	16.834%	24.644%	13.890%

**Table 3:** Model results based on 20% testing data.

Machine Learning Model	RFR	PLS	SVR	RT
**All features**				
**MAE**	1.164	1.136	1.064	1.136
**Selected features**				
**MAE**	1.157	1.299	1.244	1.135

On adding objective task difficulty as a feature to the RFR model, the 80% training data MAE lowered from 0.939 to 0.912, the 20% testing data MAE increased from 1.157 to 1.179 but the resulting model explained 29.347% of the variance in the data, higher by 9% from the model without objective task difficulty. The variables most important for the prediction were (in descending order of importance): blink frequency, eye height, objective task difficulty and baseline-related pupil diameter. Finally, LOSOXV was performed. The resulting testing MAE was 1.057, with a minimum testing error of 0.609 and a maximum testing error of 1.894.

### Statistical analysis: Effect of time-on-task and perceived effort on the features

**Fig 4:**
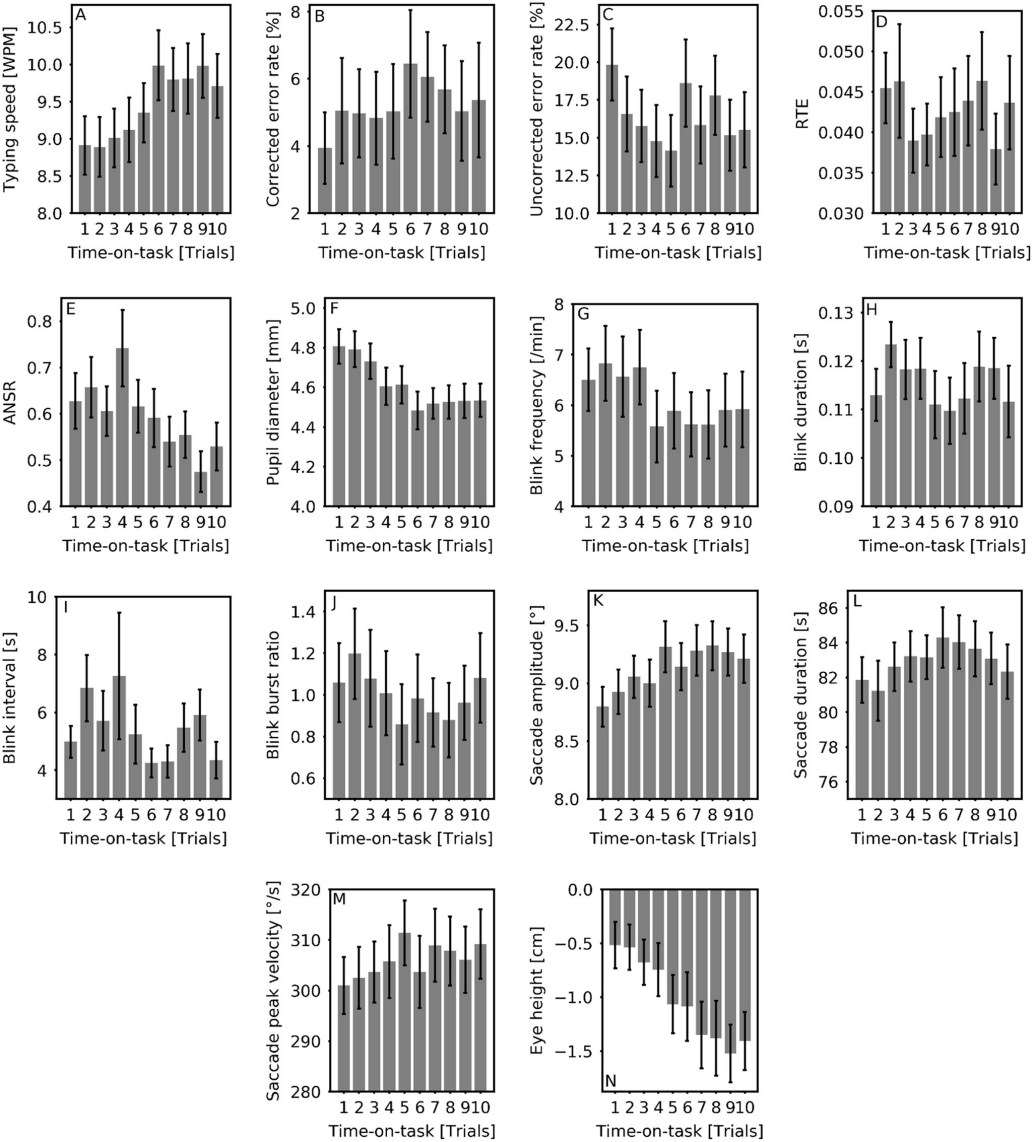
Effect of time-on-task on (a-e) performance and (f-n) eye-based features across all subjects and days for each of the 10 trials representing time-on-task. Error bars indicate standard error. RTE: Read text events ration, ANSR: Attended but not selected ratio.

To observe the impact of the relationship of mental fatigue with the perceived effort and increasing time on the features, all features were analysed using LMM, with perceived effort, time-on-task and day number as the fixed effects. The models were reduced to the optimised model for each feature, which resulted in elimination of some of the fixed effects in the end. The results of the main effects are shown in Table 4. The model coefficient *β* and its standard error are depicted in the table. A positive *β* indicates that the dependent variable increases with increasing independent variable, and a negative *β* indicates that the dependent variable decreases with increasing independent variable. The language of the experiment did not affect any of the features. The optimised model for saccade duration did not contain any fixed effect and so the feature is omitted from the table. The variation in the features with respect to time-on-task are shown in Fig 4.

**Table 4:** Linear mixed effects model results indicating the main effects of perceived effort, time-on-task and day.

Feature	Time-on-task			Perceived effort			Day number		
	*β* ± S.E.	*χ*^2^(1)	*η*^2^	*β* ± S.E.	*χ*^2^(1)	*η*^2^	*β* ± S.E.	*χ*^2^(1)	*η*^2^
**Performance features**									
Typing speed	0.126 ± 0.023	29.458^c^	0.013	-0.356 ± 0.041	69.911^c^	0.036	0.579 ± 0.062	82.969^c^	0.037
Corrected error rate	-	-	-	-	-	-	1.286 ± 0.264	22.972^c^	0.015
Uncorrected error rate	-0.405 ± 0.173	5.436^d^	0.004	5.704 ± 0.881	21.701^c^	0.245	-1.096 ± 0.466	5.506^d^	0.005
Read text events ratio	-	-	-	0.005 ± 0.001	10.598^a^	0.042	-	-	-
Attended but not selected rate	-0.019 ± 0.004	18.504^c^	0.017	0.052 ± 0.013	11.681^c^	0.046	-0.123 ± 0.012	101.23^c^	0.095
**Eye-based features**									
Baseline-related pupil diameter	-0.033 ± 0.005	43.390^c^	0.019	-	-	-	-	-	-
Blink frequency	-0.118 ± 0.046	6.463^a^	0.005	0.801 ± 0.176	14.014^c^	0.071	0.409 ± 0.124	10.701^a^	0.008
Blink duration	-	-	-	0.006 ± 0.001	35.089^c^	0.051	-	-	-
Blink interval	-	-	-	0.924 ± 0.186	23.872^c^	0.039	-	-	-
Blink burst ratio	-	-	-	0.154 ± 0.026	33.398^c^	0.031	-	-	-
Saccade amplitude	0.056 ± 0.015	13.069^c^	0.011	-0.214 ± 0.027	55.113^c^	0.056	-	-	-
Saccade peak velocity	1.042 ± 0.428	5.907^d^	0.003	-5.844 ± 0.771	54.898^c^	0.035	3.442 ± 1.144	8.987^b^	0.005
Eye height	-0.119 ± 0.024	24.672^c^	0.028	-	-	-	-0.381 ± 0.062	36.477^c^	0.041

^a^p < 0.05

^b^p < 0.01

^c^p < 0.001

^d^Bonferroni correction resulted in the feature to be non-significant (p > 0.05)

### Statistical analysis: Correlation between fatigue level and the features

Correlation to fatigue level was computed for all features. Features with an absolute correlation greater than 0.1 were: uncorrected error rate (r = 0.102, 95% CI [-0.044,0.243], p = 0.169) baseline-related pupil diameter (r = -0.178, 95% CI [-0.315, -0.034], p = 0.016), blink frequency (r = 0.184, 95% CI [0.040, 0.321], p = 0.012), blink burst ratio (r = 0.160, 95% CI [0.016, 0.299], p = 0.029) and eye height (r = -0.167, 95% CI [-0.304, -0.022], p = 0.024).

## Discussion

In the present study, we modelled mental fatigue for healthy individuals performing cognitively demanding eye-typing tasks. Cognitive load of varying degree was generated using working memory task of memorising 10 sentences of two levels of task difficulty—easy and difficult. The fatigue level showed a significant increase after each session, composed of five trials, and the terminal fatigue level was higher when the second session was difficult. The prediction of the fatigue level on a six-point Likert scale using RFR resulted in a 22% improvement from baseline MAE. On addition of objective task difficulty as a feature, the explained variance of the model increased by 9%, in comparison to the model without the feature objective task difficulty. The features selected by the final model—in decreasing order of importance were—blink frequency, eye height, objective task difficulty and baseline-related pupil diameter.

As expected, the increase in fatigue level was significant after both sessions, but only in the second session did the task difficulty have an effect on the fatigue level. Moreover, the difference between the terminal and the initial level did not depend on the order of the difficulty levels of the sessions. This indicates that there may be a non-linear relationship between task difficulty, time-on-task and mental fatigue.

The increase in the subjective fatigue level was higher after the second session (0.714) compared to after the first session (0.299). We know from literature that evaluation of the fatigue experienced can lead to re-evaluating the effort on the task and the performance generated from the effort applied [21]. This is observed in the data, as the participants evaluated their fatigue level after the first session, which may have prompted them to invest more effort in the second session, regardless of the task difficulty in the second session, resulting in the perceived effort being higher in the second session, as observed during manipulation check. This in-turn may have resulted in increase in the fatigue level after the second session. At the same time, the performance features such as typing speed and ANSR improved with time-on-task, as seen in Fig 4, depicting the application of higher effort.

The ability to apply sustained effort on a task to achieve maximum performance has been termed conation [51]. This concept can help to explain a non-linear relationship between mental fatigue, task difficulty and time-on-task. Conation provides a divergence from the resource-based theory of fatigue, which delineates a limited capacity of mental resources available for tasks, and applying effort on a task reduces some of this capacity, with reduced resources available for the subsequent tasks. The Framework for Understanding Effortful Listening (FUEL) is a model based on Kahneman’s attention model [52], and can potentially be extended to mental fatigue. The model bridges the concepts of effort to motivation level and task demands, and claims that increase in task demands or motivation can result in an increase in the effort applied on the task. In this study, the re-evaluation of fatigue after the first session and conation, along with the link between effort and motivation in increased task demands from the FUEL model could explain the observed increase in perceived effort during the second session.

Prediction of the fatigue level using eye-based data has been performed as a binary classification in literature [17, 18]. However, mental fatigue classification on a continuous scale has more uses in real-life fatigue management [18]. In this study, an RFR model of fatigue level on a six-point Likert scale predicted the 20% testing data with a MAE of 1.179. If the fatigue level on the six-point Likert scale had been classified as two classes, the mean absolute error that would have resulted in a false classification would have been 1.51. In comparison, our regression model has resulted in a lower prediction error. While this could have direct applications for non-intrusively classifying mental fatigue for people with neurological disorders, who use eye-typing in daily lives, the suggested model still needs to be re-evaluated for the target population.

The addition of task difficulty to the list of features also improved the explained variance in the data by the model by 9%, compared to the model without task difficulty. Although the MAE did not improve by inclusion of the task difficulty, it was shown to be the third-most important feature in determining mental fatigue. This suggests the importance of modelling task difficulty and cognitive load in determining mental fatigue. For future applications, recognising the difficulty level of the task could improve the prediction accuracy of mental fatigue.

The best performing machine learning algorithm was RFR, a non-linear model with four features, including task difficulty. Statistical analysis methods were undertaken to understand the working of the machine learning model. Two of the four features—baseline-related pupil diameter and eye height, showed a linear effect of time-on-task and correlated to the subjective fatigue level with absolute correlation value of greater than 0.1. The third, and the most important feature selected—blink frequency—not only showed effects of time-on-task and correlated to the fatigue level, but also showed high effects of the perceived effort.

The feature blink burst ratio showed conflicting effects of time-on-task and correlation with fatigue level, compared to blink frequency. While blink frequency reduced with time-on-task, blink burst ratio did not show any effect of time-on-task. Although, both the features depicted positive correlation with the fatigue level, only blink frequency was selected by the RFR model. The only other difference between the features was that blink frequency also showed effects of the perceived effort. Blink frequency was selected to be the most important feature by the model. This working of the model indicates that fatigue level might be controlled by both time-on-task and perceived effort.

The generally low variance explained by the machine learning model (30%) can be attributed to this complex nature and relations of mental fatigue, time, cognitive load and possibly other related variables such as motivation, circadian rhythm and food and caffeine intake [1, 10, 53], which were not controlled for or included in the scope of this study.

The LMM showing the effect of perceived effort, time-on-task and day number on the features suggest that several features did not behave as expected, with respect to time-on-task. As per the model coefficient values (*β*), blink frequency reduced with increasing time-on-task while saccade amplitude and saccade peak velocity increased. A possible explanation for the blink frequency could be the increase in the effort applied, indicated by the perceived effort, during the second session, as the participants attempted to concentrate more while performing the second session and thereby, blinked less often. Another explanation could be that the increase in the effort applied could be accompanied by an increase in the arousal level, resulting in increase in saccade peak velocity [54], and thereby increased saccade amplitude as time-on-task increased. The features studied in the study have not been previously studied in combination with an interactive eye-based task, and the effect of such an interaction may have affected the behaviour of the features in response to cognitive load and time-on-task.

All the features analysed using LMM resulted in the perceived effort having a stronger effect on the features than time-on-task. The balancing order of the difficulty levels on different days could have reduced the average effect of time-on-task over each day.

There are additional limitations in this study. No objective measurement of fatigue was conducted, using e.g. attention tests [55], which could have confirmed the subjective fatigue level. Although the participants performed two trials for practice, the eye-typing task was not a common task in the participants’ everyday lives, and there was a large learning effect over the days. Finally, all the features examined within the study, with the exception of baseline-related pupil diameter and eye height, are known to be affected by cognitive load, which may further have resulted in unexpected variations in the features, such as improvement in performance features with time-on-task.

## Conclusion

Results from the current study indicated that mental fatigue prediction as a regression problem has a feasible solution. Moreover, mental fatigue, perceived effort and time-on-task are inter-linked in a complex manner, and modelling of mental fatigue depends on both time-on-task and perceived effort. We were able to successfully make reasonable predictions of the fatigue level using three eye-based features, during an eye-typing task—blink frequency, eye height and baseline-related pupil diameter. On including task difficulty as an additional feature to predict the fatigue level, the variance explained by the machine learning RFR model improved. These results are a step towards a better understanding of the cognitive state of mental fatigue. Finally, it contributes to the development of a non-intrusive method for continuous mental fatigue detection, that could benefit both people with neurological diseases and general working population.

## Supporting information

S1 Fig Effect of the perceived difficulty on the features. (TIF)

S2 Fig Effect of the day number on the features. (TIF)
