# Supplementary figures and images for: Mental fatigue prediction during eye-typing
Source: PLoS One. 2021 Feb 22;16(2):e0246739. doi: 10.1371/journal.pone.0246739 (PMC7899326; doi:10.1371/journal.pone.0246739)

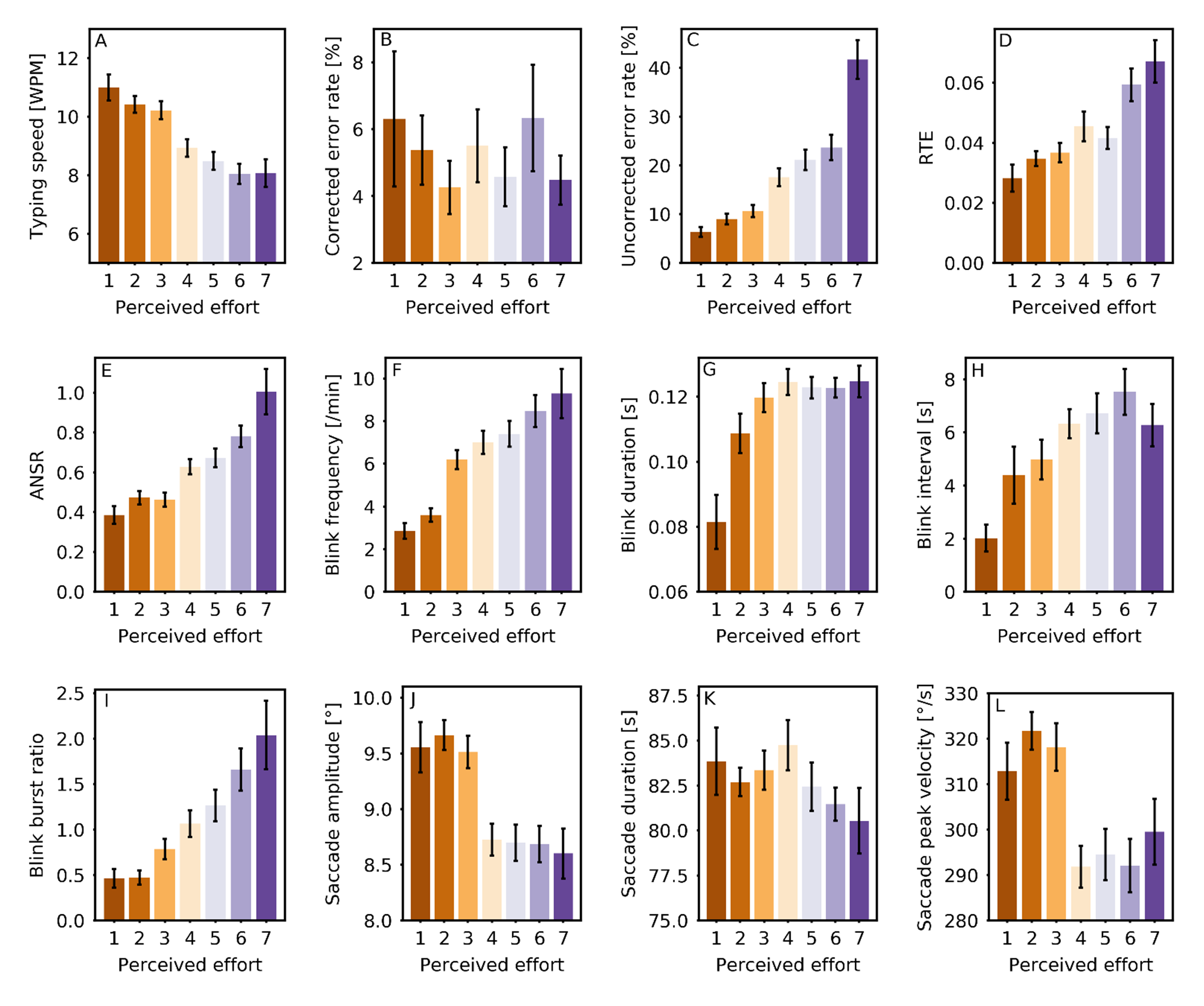

Supplement: S1 Fig — (TIF) [file pone.0246739.s001.tif]

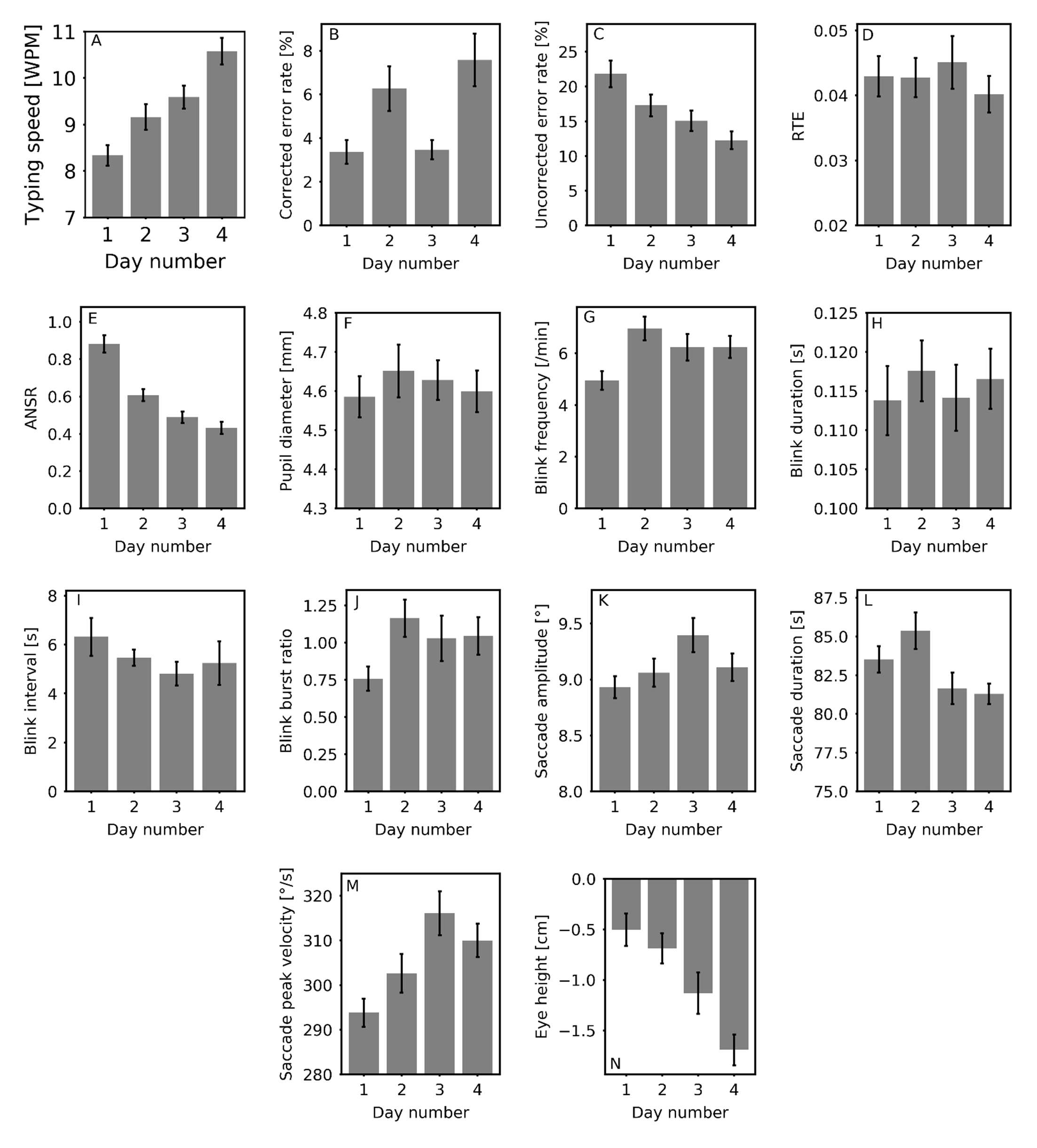

Supplement: S2 Fig — (TIF) [file pone.0246739.s002.tif]
